# Evaluation of the color-matching potentials of different universal composite resins with natural tooth structure in class III through-and-through cavities using a visual guide and spectrophotometer

**DOI:** 10.34172/joddd.025.42343

**Published:** 2025-12-31

**Authors:** Mahmoud Bahari, Mahdi Abed Kahnamouei, Fatemeh Pournaghi Azar, Soodabeh Kimyai, Kiana Tadayon

**Affiliations:** ^1^Dental and Periodontal Research Center, Tabriz University of Medical Sciences, Tabriz, Iran; ^2^Department of Esthetic and Restorative Dentistry, Faculty of Dentistry, Tabriz University of Medical Sciences, Tabriz, Iran

**Keywords:** Color, Composite resin, Esthetics, Resins, Spectrophotometry

## Abstract

**Background.:**

This study assessed the color-matching potentials of Omnichroma single-shade composite resin and Omnichroma Blocker with natural tooth structure in Class III through-and-through restorations and compared them with a universal multi-shade composite resin using both visual and spectrophotometric evaluations.

**Methods.:**

Class III through-and-through cavities were prepared on the mesial and distal aspects of 32 extracted anterior teeth. Each preparation was restored with either Omnichroma composite resin or Tetric Evoceram composite resin, with or without an 0.5-mm depth of Omnichroma Blocker on the lingual aspect. Color measurements were performed initially and after restoration using visual evaluation with the VITA Classical Shade Guide and the SpectroShade microspectrophotometer. Color differences were calculated using the CIEDE2000 formula.

**Results.:**

Tetric Evoceram with and without blocker exhibited a statistically significant difference in color match with natural tooth structure (*P*=0.02). The color-matching difference with natural teeth was statistically significant in the visual evaluation (*P*=0.005).

**Conclusion.:**

Failing to use a blocker with Tetric Evoceram composite resin in large Class III cavities reduced color matching. In contrast, using a blocker with Omnichroma composite resin did not affect the final color-matching ability. Furthermore, the Omnichroma composite resin demonstrated better visual color matching, regardless of the presence of a blocker, than the Tetric Evoceram composite resin.

## Introduction

 Dental composite resins are currently among the most commonly used restorative materials worldwide.^1,2^ Composite resins are available in various colors and translucencies, including dentin, opaque, and enamel (or translucent) options, which restore the optical properties of dentin and enamel.

 Recent developments in adhesive dentistry have enabled composite resin restorations to significantly replicate the function and appearance of natural tooth structures.^3^ Despite the progression of these materials, color matching of composite resins with natural tooth structure remains one of the most complex and critical aspects of direct composite resin restorations, particularly in the anterior region, as it reflects the dentist’s professional expertise and skill.^4-6^ The need for simplified color selection has led to the development of universal composite resin materials designed to match all tooth colors, simplifying the restoration process and minimizing clinical errors.^7,8^ Manufacturers may employ different color parameters for composite resin materials, reflecting compositional differences that can affect light absorption and scattering properties.^9^

 Omnichroma composite resin (Omnichroma, Tokuyama Dental, Tokyo, Japan), introduced in 2019, is a single-shade universal composite resin that utilizes structural color technology. According to the manufacturer, it matches all Vita Classic Shade Guide colors. Structural color is produced solely through the physical properties of light, such as refraction, interference, and scattering, without the exchange of light energy. To achieve this structural color, the composite resin fillers consist of spherical particles of uniform size (260 nm) that function as an additive color-mixing system.^10-12^

 When ambient light passes through the composite resin, these micron-sized spherical particles can produce a red-to-yellow color, which is combined with reflected light and the color of the tooth structure through additive color mixing, enhancing Omnichroma’s ability to match natural teeth.^7^ In Class III and IV through-and-through lesions, color matching can be challenging due to insufficient tooth structure and the darkness of the oral cavity beyond the translucent material. According to the manufacturers, Omnichroma blockers are used as supplementary materials in the form of a lingual layer to effectively reduce the darkness of the oral cavity.^10-12^

 There are two methods for measuring color: instrumental and visual. The instrumental approach uses a spectrophotometer to determine the amount and spectral composition of light reflected from an object and then converts this data into measurable values. Spectrophotometers are considered more reliable than colorimeters because they are not affected by the metamerism of objects.^13-16^

 Spectrophotometers quantify color using standardized systems such as CIELab (L*a*b*), where L* represents lightness, a* is the red-green axis, and b* is the yellow-blue axis. The ΔE (color difference) value is often calculated to determine how distinguishable two colors are, ensuring accuracy in shade matching for crowns, veneers, and composite resin restorations.^17^

 CIELab and CIEDE2000 are recommended by the International Commission on Illumination (CIE) for evaluating color differences (ΔEab and ΔE00, respectively). Although CIEDE2000 is more sophisticated and its computational procedure is more complex, potentially limiting its applicability, it correlates much better with visual findings. Therefore, CIEDE2000 was used in this experiment.^18^ While a spectrophotometer can accurately provide the specifics of color and its intensity, visual perception remains critical in patient acceptance.^19,20^

 Previous studies have investigated the color-matching potential of these composite resins on acrylic molar denture teeth, yielding conflicting results.^21^ However, the color-matching capacity of this material and its ability to compete with pigmented-colored universal composites have yet to be extensively investigated in anterior restorations on natural teeth.

 Given the clinical importance of simplifying color matching in anterior restorations and the potential of Omnichroma universal composite resin to facilitate this goal, as well as the limitations of existing data on the optical properties and color matching of this composite resin and its blocker in extensive anterior restorations on natural teeth, this study aimed to investigate the color matching ability of two different universal composite resins with natural tooth structures in Class III through-and-through cavities using both a visual guide and a spectrophotometer.

 The following null hypotheses were tested:

1. There is no color difference between Omnichroma single-shade universal composite resin, with or without a blocker, and the tooth structure in Class III through-and-through cavities, as assessed by visual and spectrophotometric evaluation.
2. There is no color difference between the Tetric Evoceram composite resin, with or without a blocker, and the tooth structure in Class III through-and-through cavities, as assessed by visual and spectrophotometric evaluation.
3. There is no color-matching difference between the Omnichroma single-shade universal composite resin and Tetric Evoceram composite resin, with or without a blocker, and the tooth structure in Class III through-and-through cavities, as assessed by visual and spectrophotometric evaluation.

## Methods

 To determine the sample size, based on the study by Peter Mourouzis, the mean (standard deviation) of color changes in the OC and TE groups were 4.08 (0.85) and 2.13 (0.85), respectively. Considering a type I error of 0.05 and a power of 80%, 6 samples per group were calculated. To increase the study’s reliability, the sample size was increased by 20%, resulting in 8 samples per group.^22^

 This in vitro study used 32 freshly extracted, intact human anterior teeth. The teeth were visually examined and probed, and only those without caries, cracks, previous restorations, or discoloration were selected. The selected teeth were cleaned using a rubber cup and pumice and subsequently stored in distilled water.^22^

 The initial color of each tooth was first determined by visual evaluation using the 16 colors (A1-D4) of the Vitapan Classical shade guide (Vita, Bad Säckingen, Germany), which was placed in contact with the incisal edge of the teeth. Observers had only 25 seconds to look at each sample. After determining the color, they immediately placed the sample back into the water container and moved on to the next one.

**Figure 1:**
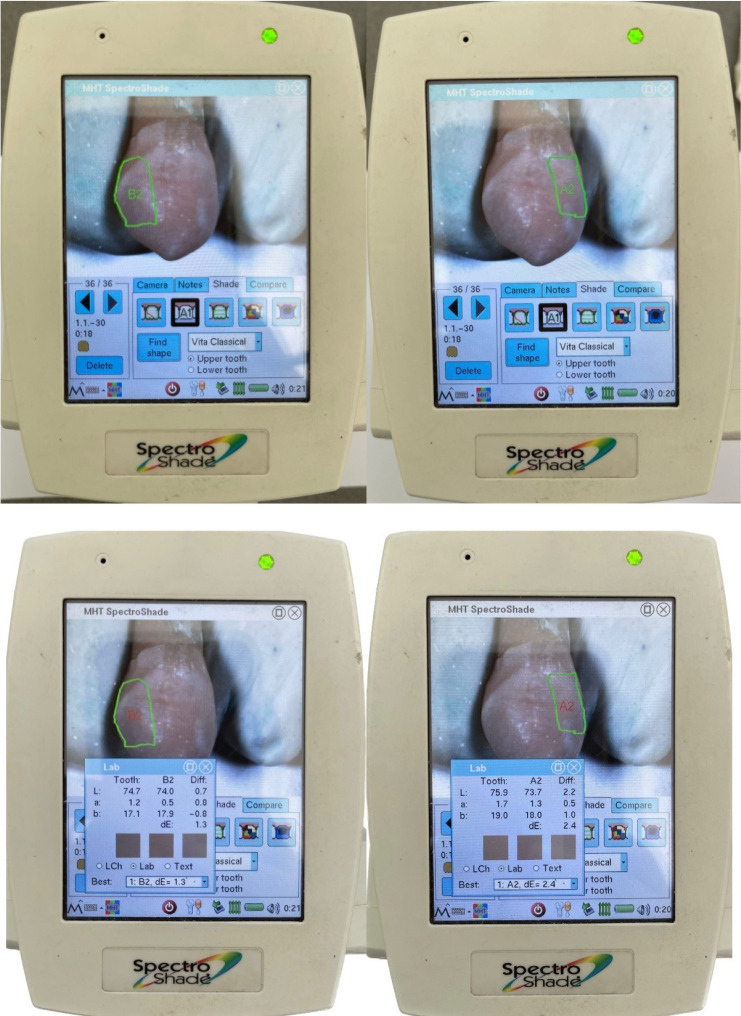
Initial color measurements with a spectrophotometer

 To confirm the selected shade, the initial color was finalized, and spectral reflectance values were recorded. The SpectroShade Micro spectrophotometer (SpectroShade Micro, MHT S.P.A., Milan, Italy) was used on the buccal surface, with three consecutive readings taken for each sample (Figure 1). The L*, a*, and b* parameters were recorded, and the average readings were calculated. The samples were immediately returned to their respective water container after evaluation.^7,23,24^

 Then, two impressions of each tooth were made using the double impression technique with additional silicone impression materials (Panasil Tray Heavy and Panasil Initial Contact Light; Kettenbach GmbH & Co., Eschenburg, Germany). The first impression was a silicone matrix to recreate the original tooth anatomy. The second impression served as a mold, allowing repeated measurements with the spectrophotometer at the same position as during initial calibration.^7,22,23^

**Figure 2:**
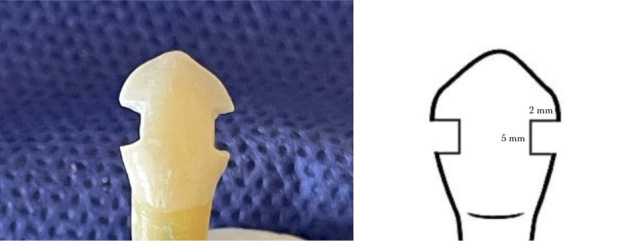
Class III through-and-through preparations on mesial and distal aspects

 Class III through-and-through preparations were created on the mesial and distal aspects of each tooth, with an axial depth of 2 mm and an incisogingival length of 5 mm, using an 008 diamond bur with water (Komet, Germany) (Figure 2). The bur was replaced after preparing four cavities. The preparations were thoroughly rinsed with water and air-dried. Then, the surfaces were acid-etched with 37% phosphoric acid gel (Eco-Etch, Ivoclar Vivadent, Liechtenstein) for 20 seconds, rinsed with a water spray, and air-dried. Adper^TM^ Single Bond 2 Adhesive (3M ESPE, St. Paul, MN, USA) was applied according to the manufacturer’s instructions and polymerized using an LED light-curing device (Woodpecker, Guilin, China).^25^

 The radiation output of the light-curing device was continuously checked with a dental radiometer (Coltolux Light Meter (Coltene-Whaledent, Sussex, UK)) before being used for each group.^7^

**Figure 3:**
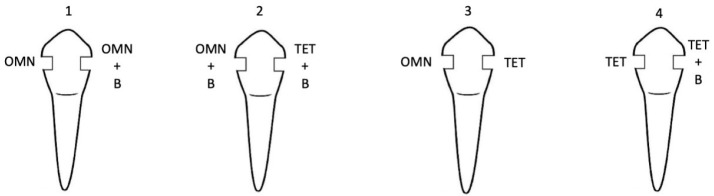
Study groups. OMN: Omnichroma composite resin (Omnichroma, Tokuyama Dental, Tokyo, Japan) TET: Tetric Evoceram composite resin (Ivoclar Vivadent Schaan, Liechtenstein) B: Omnichroma Blocker composite resin (Omnichroma, Tokuyama Dental, Tokyo, Japan.

 Then, the samples were randomly divided into four groups (n = 8) according to the composite resin used. These composite resins included Omnichroma and its blocker, Omnichroma Blocker, and Tetric Evoceram. (Figure 3; Table 1).

**Table 1:** Materials Used and Their Main Components

**Material**	**Specifications**	**Composition**	**Manufacturer**
Omnichroma	light-cured, radiopaque single shade universal composite	The Filler System: 79% by weight (68% by volume) of spherical silica- zirconia filler (mean particle size: 0.3 μm) and composite filler. The Resin System UDMA, TEGDMA -Mequinol, Dibutyl hydroxyl toluene and UV absorber.	Tokuyama Dental, Tokyo, Japan
Omnichroma Blocker	light-cured, Spra-nano filled, radiopaque single shade universal composite	The Filler System: 82% by weight (71% by volume) of spherical silica- zirconia filler (mean particle size: 0.2 μm) and composite filler. The Resin System: Bis-GMA, triethylene glycol, dimethacrylate	Tokuyama Dental, Tokyo, Japan
Tetric EvoCeram	light-cured, radiopaque, nanohybrid, Multi shade universal composite	The Filler System: 79% by weight (60% by volume) Barium glass, silica dioxide, ytterbium trifluoride, barium alumino- fluorosilicate glass. (mean particle size: 0.7 μm) The Resin System: Bis-GMA, Bis-EMA, UDMA	Ivoclar Vivadent, Amherst, NY

UDMA, Urethane dimetacrylate; TEGDMA, Triethylene glycol dimethacrylate; Bis-GMA, Bisphenol A diglycidylmethacrylate; Bis-EMA, Bisphenol A polyethylene glycol diether dimetacrylate

**Figure 4:**
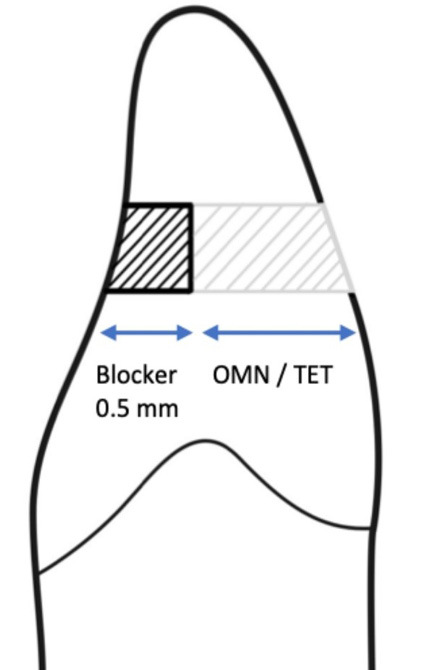
Composite resin restoration layers

 Simple randomization was performed using random numbers generated by Randlist Software version 1.2 (Randlist Inc., Chicago, USA) to allocate the samples to the study groups. The silicone putty index was first divided into two halves: lingual and buccal, for placing the composite resin restorations. The lingual half was placed on the tooth, and a layer of composite resin was applied to form the lingual shell (Figure 4).

 The cavity margins were prepared without a bevel. The samples were restored with composite resin in the color determined by the spectrophotometer. Each composite resin layer was applied with a thickness of 1 mm and light-cured for 30 seconds. The focus was on reconstructing the original tooth anatomy using the silicone matrix.^23,26^

 To replicate the anatomy of intact teeth in the composite resin restorations, a polytetrafluoroethylene film layer was placed over the non-polymerized final composite resin layers, and the buccal half of the silicone putty index was pressed onto it. After removing the polytetrafluoroethylene film, the composite resin restorations were polymerized for 40 seconds using an LED light-curing device, following the manufacturer’s instructions.^7^

 A series of diamond discs (Soflex Pop-On, 3M Oralcare) was used for finishing and polishing, followed by rubber points and cups to remove the oxygen-inhibited surface layer from the lingual surface. The samples were then stored in distilled water for 48 hours before color measurement procedures. A single operator conducted all procedures.^13^

**Figure 5:**
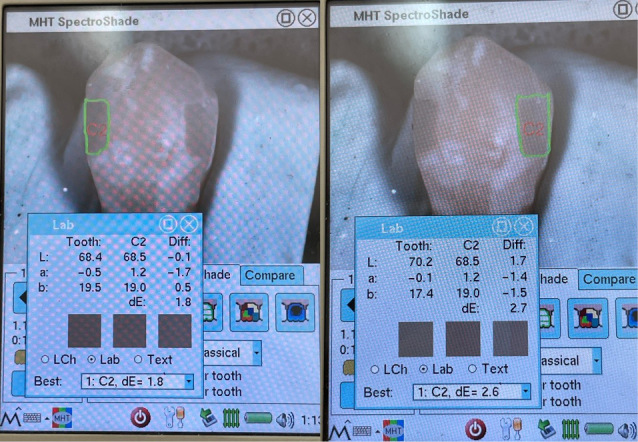
Post-procedural color measurements with a spectrophotometer


 Spectrophotometric evaluation was conducted on the restorations on each tooth’s mesial and distal aspects using the indices prepared before the intervention to calibrate the position. Three consecutive readings were taken for each restoration, and the average parameters L*, a*, and b* were recorded (Figure 5). The total color difference between the intact tooth and each restoration was calculated using the CIEDE2000 formula,

$$\Delta E_{00}=\sqrt{{\left(\frac{\Delta L^{\prime }}{k_{L}s_{L}}\right)}^{2}+{\left(\frac{\Delta c^{\prime }}{kc^{S}c}\right)}^{2}+{\left(\frac{\Delta H^{\prime }}{k_{H}s_{H}}\right)}^{2}+R_{T}\left(\frac{\Delta c^{\prime }}{k_{c}S_{c}}\right)\left(\frac{\Delta H^{\prime }}{k_{H}s_{H}}\right)}$$

where ΔL is the change of lightness, ΔC = difference in chroma, ΔΗ = difference in hue; and Rt, Sl, Sc, and Sh are weighting factors. The 3 K terms are additional weighting factors that were set to 1.^18^

 Visual color-matching evaluation of the restoration and the tooth, and determination of the color of each restoration was performed by six observers (four women and two men), all restorative dentistry residents with normal color vision, as verified by the Ishihara test.^27^

 Each sample was evaluated against a neutral gray background at a distance of 25 cm and an angle of 45° under natural light in summer, on one day from 10 am to 2 pm, by the same window facing north, with indirect sunlight (to avoid glare) and a clear sky.

 The observers were given 25 seconds to evaluate each sample. After determining the color match between the teeth and the restorations on the mesial and distal aspects of each tooth using the Vita Classical Shade Guide, they placed the sample back in the water container and moved on to the next one. Each observer repeated this process for teeth #1 to #32, and the entire procedure was completed in a single day. The color difference between the restoration and the surrounding tooth structure was rated on a scale from 0 to 4:

 0: excellent match

 1: very good match

 2: acceptable match

 3: obvious mismatch

 4: high mismatch

 After evaluating each sample, the observers were allowed to view a neutral gray background to prevent eye fatigue.^7,22^

 Inter-observer agreement was evaluated to assess the consistency of ratings between observers. Fleiss’ kappa, a statistical measure of agreement for categorical data involving more than two raters, was used because it accounts for agreement that occurs by chance.

 Intra-observer agreement was determined as the mean value of the highest percentage of identical scores a particular observer gave for six samples of the same color (i.e., for each observer).

 The normality of the data was assessed using the Kolmogorov–Smirnov test and a histogram chart. The Wilcoxon test was used to compare the mean differences in color matching between the two groups because the variables were non-normal. The Kruskal-Wallis test, a nonparametric method, was used to compare mean differences in color matching between the four groups, as all variables (mean differences in tooth color matching in the four groups) exhibited non-normal distributions. If the Kruskal-Wallis test results were significant, a post hoc Dunn test was conducted to identify specific groups with statistically significant differences in mean matching differences. A *P*-value < 0.05 was considered statistically significant. Data analysis was performed using Stata version 16.

## Results

Table 2 shows the average color-matching differences of the materials used with natural teeth across different intervention groups, evaluated visually or with a spectrophotometer. In the spectrophotometric evaluation, the highest mean color difference compared to natural teeth was observed in the Tetric group without a blocker (5.01 ± 1.08), while the lowest was in the Tetric group with a blocker (4.06 ± 1.30).

**Table 2:** Comparison of the average color difference with natural teeth in different intervention groups

**Variable**	**Spectrophotometric Evaluation**		***P*** -value*	**Visual Evaluation**		***P*** -value*
	**Mean** **ΔE00**	**Standard Deviation**		**Mean**	**Standard Deviation**	
Color difference of OMN^1^ without B^2^ compared to natural tooth	4.29	0.94	0.66	0.8	0.57	0.44
Color difference of OMN with B compared to natural tooth	4.40	1.28		0.60	0.51	
Color difference of TET^3^ without B compared to natural tooth	5.01	1.08	0.02	2.27	0.99	0.09
Color difference of TET with B compared to natural tooth	4.06	1.30		1.83	0.82	

OMN: Omnichroma Composite (Omnichroma, Tokuyama Dental, Tokyo, Japan) TET: Tetric Evoceram Composite (Ivoclar Vivadent Schaan, Liechtenstein) B: Omnichroma Blocker Composite (Omnichroma, Tokuyama Dental, Tokyo, Japan

 In the visual evaluation, the lowest average color difference was found in the Omnichroma group with a blocker (0.6 ± 0.51), and the highest was in the Tetric group without a blocker (2.27 ± 0.99). Among the compared groups, only the mean difference in color matching between the Tetric without blocker and Tetric with blocker groups, as assessed by spectrophotometry, was statistically significant compared to natural teeth (*P* = 0.02).

 The results of the Kruskal-Wallis test revealed a statistically significant difference in color match scores between the groups in the visual evaluation (*P* = 0.005). However, the test did not reveal a statistically significant difference in the spectrophotometric evaluation (*P* = 0.49). These findings indicate that while the visual assessment identified differences between the groups, the spectrophotometric evaluation did not detect significant variations. A post hoc Dunn test was conducted to identify differences between specific groups.

 Dunn’s post hoc test revealed that the color-matching difference for the Tetric Evoceram composite resin without a blocker significantly differed from that of Omnichroma without a blocker (*P* = 0.004) and with a blocker (*P* = 0.0006). Additionally, the color-matching difference between Tetric Evoceram with a blocker and Omnichroma with a blocker was significantly different (*P* = 0.018).

 The consistency between observers was assessed using Fleiss’ Kappa. Before the intervention, the inter-observer agreement was 51.67% (Fleiss’ kappa = 0.52), indicating a moderate level of agreement. After the intervention, the agreement decreased slightly to 49.69% (Fleiss’ kappa = 0.50), representing moderate agreement.

 Intra-observer agreement was assessed using the percentage of agreement, calculated as the percentage of trials in which the observers’ ratings matched the true category (A1). Each observer rated the same sample six times, and agreement was computed as the proportion of correct ratings across all trials.

 The percentage of agreement for individual observers was as follows: Observer 1 (66.67%), Observer 2 (83.33%), Observer 3 (66.67%), Observer 4 (66.67%), Observer 5 (83.33%), and Observer 6 (66.67%).

## Discussion

 Monochromatic composite resins have recently been introduced to address patients’ increasing desire for natural-looking restorations. These composite resins are designed to match all VITA Classic A1-D4 shades using a single shade by reflecting specific wavelengths corresponding to the tooth color.^6^ According to the manufacturer, Omnichroma does not contain pigments; its color properties are based on structural color. This is achieved through smart color technology that controls the optical properties of the composite resin.^23^

 Laboratory and clinical research has been conducted to understand the optical behavior of composite resins and their interaction with tooth structures. These studies aim to develop accurate color measurement protocols and determine the color match between restorations and intact tooth structures.^11^

 In the present study, the color difference between Omnichroma single-shade universal composite resin, with or without Omnichroma blocker, and tooth structure in Class III through-and-through cavities was not statistically significant, as evaluated by visual and spectrophotometric methods. Therefore, the first research hypothesis was not supported.

 Following the manufacturer’s instructions, this study used an 0.5-mm-thick Omnichroma Blocker in the lingual portion. However, it has been reported that the translucency of Omnichroma Blocker varies with different thicknesses, and this material does not effectively cover the background with thicknesses of 0.5 to 1 mm. This suggests that the thickness used in the current study may have been insufficient, resulting in no significant difference compared to not using the blocker.^10^

 Additionally, the teeth used in this study were primarily maxillary central incisors and canines with a high buccolingual thickness. Consequently, the Omnichroma composite resin layer used for extensive Class III restorations was relatively thick. A previous study stated that as the thickness of composite resins increases, their translucency decreases. This could explain the lack of a significant color difference in cavities restored with Omnichroma composite resin, whether or not a blocker was used.^6^

 In the current study, the color difference between Tetric Evoceram multi-shade universal composite, with or without a blocker, and the tooth structure in Class III through-and-through cavities was found to be statistically significant by spectrophotometric evaluation (P = 0.02), as determined by the Wilcoxon test. Tetric Evoceram with a blocker had the lowest mean color difference among the four groups. In contrast, Tetric Evoceram without a blocker had the highest mean color difference. Therefore, the null hypothesis stating that there is no color difference between Tetric Evoceram without a blocker and the tooth structure in Class III through-and-through cavities was rejected based on spectrophotometric evaluation.

 According to the literature, the Tetric Evoceram composite resin exhibits the highest level of translucency among the materials studied. Consequently, it is expected that using a blocker layer can facilitate color matching with the adjacent tooth structure.^12^ However, in this study, the difference in color between Tetric Evoceram composite resin with or without a blocker in Class III through-and-through cavities and the tooth structure was not statistically significant by visual evaluation. Therefore, the second research hypothesis was accepted based on the visual evaluation.

 These findings are consistent with a previous study, which demonstrated that visual color evaluation showed a low-to-moderate correlation with spectrophotometric evaluation.^17^

 According to previous studies, visual color determination is a complex process influenced by subjective and objective factors. The inconsistency in results between different observers and even with the same observer, combined with the variability in human sensitivity to hue, chroma, and value, contributes to the unreliability of this method despite the standardization of observation conditions.^7,15,20^

 Compared to visual color selection under standard conditions, a spectrophotometer is more accurate in matching the original color. As a result, spectrophotometers are currently regarded as one of the most accurate and valuable instruments for color determination.^14,15^ According to the literature, the SpectroShade Micro spectrophotometer used in this study has the best repeatability and accuracy among the available spectrophotometers.^5^

 In this study, the Kruskal-Wallis and post hoc Dunn tests revealed that the color matching of Tetric Evoceram composite resin without a blocker was significantly different from the color matching of Omnichroma composite resin with a blocker (P = 0.004) or without a blocker (P = 0.006) in Class III through-and-through cavities. Moreover, Omnichroma demonstrated better color matching. This finding is consistent with a previous study, highlighting that Omnichroma composite resin, with its supra-nano spherical fillers, achieves superior color matching with surrounding dental structures through structural color stimulation.^2^

 Additionally, it was observed that on extracted teeth, Omnichroma monochromatic composite resin effectively matched the color of the surrounding tooth structure. However, the color of the Omnichroma composite resin in the restored tooth differed significantly from that of the original composite resin discs. This indicates that while Omnichroma is compatible with the surrounding tooth structure, it can change color from its initial hue.^23^

 In this study, the Kruskal-Wallis and post hoc Dunn tests revealed that the color matching of Tetric Evoceram composite resin with a blocker in Class III through-and-through cavities was significantly different from that of Omnichroma with a blocker by visual evaluation (P = 0.018), whereas Omnichroma showed insignificant color matching by spectrophotometric evaluation. Therefore, the third hypothesis of this study was accepted by visual evaluation but rejected by spectrophotometric evaluation. This finding contrasts with a previous study, which reported that Omnichroma composite resin showed a greater color difference, both visually and spectrophotometrically, while Tetric Evoceram had a better color match.^7^

 This discrepancy may be attributed to differences in study design. The referenced study used acrylic molar teeth with Class I cavities, which allowed for structural color reflection from the remaining tooth structure. In contrast, the current research focused on anterior natural teeth with extensive Class III through-and-through cavities, in which the lingual tooth structure was completely removed. Additionally, this study employed the CIEDE2000 formula, which, despite its complexity, has shown better consistency with visual findings and can detect color differences more discernible to the human eye. The CIEDE2000 formula is also more sensitive to color changes than the CIELAB formula (ΔEab) used in the referenced study.^8^

 This finding contradicts one study, which reported better color matching with Tetric Evoceram composite resin than Omnichroma. However, they used posterior acrylic teeth and employed a visual assessment scoring scale with only three values, but we used the five-value scale in the current study.^13^

 According to previous studies, the perceptibility threshold (PT) and clinical acceptability threshold (AT) for ΔE{00}have been reported to be approximately 1.8 and 2.25, respectively. As shown in Table 2, all mean* ΔE*{00} values obtained in the present study were higher than those for both PT and AT. This indicates that the color differences observed between the tested groups were not only statistically significant but also clinically perceptible and unacceptable, reflecting a visible color mismatch.^4^

 The absence of saliva and the vitality of the teeth represent methodological limitations of this in vitro study, hindering its ability to replicate clinical conditions. Moreover, healthy extracted teeth were used in this study, and the ability of this composite resin to match the color of discolored or stained teeth due to various causes was not assessed. Additional research is recommended to evaluate the color-matching ability of Omnichroma composite resin across a broader range of tooth shades and cavity preparations.

 To promote future research, we suggest conducting clinical evaluations of vital teeth in the oral cavity, assessing a broader range of tooth shades, and examining different cavity sizes and depths. It would also be valuable to study the composite’s blending and chameleon properties and the impact of both in-office and at-home bleaching treatments on its color-matching ability.

 However, this study faced limitations due to its laboratory setting with extracted teeth, which could not fully replicate clinical conditions. For instance, varying restoration depths, specific clinical contexts of individual teeth, the presence of saliva, and the teeth’s natural vitality were not considered. Additionally, the study used a few healthy extracted teeth, and the composite’s ability to match the color of discolored or stained teeth was not evaluated. Therefore, further research is needed to assess these materials’ in vivo predictability using natural teeth and to explore a broader range of shades.

 While natural daylight is considered the best source for color matching, its variability can introduce inconsistencies despite efforts to standardize lighting conditions.

 The Omnichroma Blocker layer was limited to 0.5 mm according to the manufacturer’s instructions, whereas thicker layers (0.7–1.0 mm) reported in the literature may provide better masking of dark backgrounds.

## Conclusion

1. Based on this study’s findings, the use of a blocker at the manufacturer’s recommended thickness (0.5mm) with Omnichroma composite resin does not affect the final color matching.
2. In extensive Class III cavities, the lack of a blocker in the Tetric Evoceram composite resin leads to diminished color matching, which can only be identified through spectrophotometric analysis.
3. Omnichroma composite resin demonstrated superior visual color matching compared to Tetric Evoceram, both with and without a blocker. When a blocker was used with either composite resin, Omnichroma showed better color matching as assessed by visual evaluation of the tooth structure.

## Competing Interests

 The authors declare that they have no financial or non-financial competing interests related to the publication of this work. If any potential competing interests arise, we will promptly inform the editorial office in accordance with the journal’s policies.

## Ethical Approval

 The study protocol was approved by the Regional Medical Research Ethics Committee (IR.TBZMED.REC.1401.603).
